# Erector spinae plane blocks for day-case medical thoracoscopy: a pilot clinical study

**DOI:** 10.1515/pp-2022-0115

**Published:** 2022-11-15

**Authors:** Jamie McPherson, Edward Halvey, Avinash Aujayeb

**Affiliations:** Anaesthetic Department, Northumbria Healthcare NHS Trust, Newcastle, UK; Respiratory Department, Northumbria Healthcare NHS Trust, Newcastle, UK

**Keywords:** erector spinae block, local anaesthetic thoracoscopy, medical thoracoscopy, nerve block

## Abstract

**Objectives:**

Erector spinae plane (ESP) blocks are a regional anaesthetic technique used for pain relief in thoracic procedures. Our centre has recently begun using ESP blocks pre-medical thoracoscopy for analgesia.

**Methods:**

Nine patients undergoing MT from September 2021 to February 2022 were included. Opioid use and depth of required sedation was recorded. Pre and post pain scores and at home were recorded by interview and review of charts. A functional pain questionnaire was administered via telephone.

**Results:**

Average greatest depth of sedation using propofol was 1.92 (standard error of mean [SEM] 0.27), with remifentanil 2.52 (SEM 0.46). 78% required oral analgesia on day 0 post discharge. 55% required oral analgesia on post-op day 1. Patients used an average of 3.33 mg oral morphine (SEM 2.35) in hospital, and 3 mg (SEM 2) on post-op day 1. Periprocedural pain scores were 0.66 (SEM 0.27). Pain scores in recovery were 1.56 (SEM 0.76). Pain scores 3–12 h post discharge were 3.56 (SEM 0.7), while pain scores on post-op day 1 were significantly higher at 5.56 (SEM 0.90) (Figure 1). Functional pain scoring showed patients doing activities of daily living well with a good ability to breathe and cough. All felt that their pain was well controlled on the day of the procedure and at home. No complications were reported.

**Conclusions:**

ESP blocks provide good analgesia. Pain scores showed significant analgesic effect lasting several hours. The project showed pain outcomes and patient acceptability were good for the use of regional anaesthesia.

Established guidance for pain management for video-assisted thoracoscopic surgery (VATs) exists [1]. A VAT procedure involves general anesthesia, single lung ventilation and the insertion of a port into the affected pleural space to drain fluid, biopsy tissue and prevent recurrence of fluid by performing pleurodesis or manage any recurrence by placing an indwelling pleural catheter. However, over the course of the last few years, medical thoracoscopy (MT) or local anaesthetic thoracoscopy has established itself as a safe procedure for the investigation and treatment of pleural effusions with high diagnostic sensitivity (comparable to VATS) and is performed by interventional respiratory physicians [2, 3]. In the United Kingdom (UK), direction is available from the now outdated British Thoracic Society (BTS) 2010 guidelines which summarized evidence from 361 patients [4]. The main differences between MT and VATS are that during MT, the patient is sedated, is breathing spontaneously and an artificial pneumothorax is created by allowing air to enter the pleural space and only one port is inserted through which a rigid or a semi-rigid scope can be passed through [5]. However, the surgical trauma and resultant pain stimulus are highly comparable to VATs: discomfort associated with the procedure can be significant, leading to difficult operating conditions and pain afterwards [4, 5]. There is significance variance on how MT is performed and what pain management is appropriate. Various strategies have been used, from conscious sedation by combining a benzodiazepine with opioids or titrated propofol infusions with or without opioids, insertion of erector spinae or paravertebral blocks or intrapleural lignocaine installation [3], [4], [5]. Most of the studies are cohort studies, or single centre randomised controlled trials with small patient numbers with the potential from bias from their open-label nature. Unpublished data from our previously described cohort [2] which were all previously admitted suggested high opiate use post-operatively for pain and this often led to a few days of inpatient stay, with a reported median length of stay of 3.96 days.

Feray et al. [1] suggest that erector spinae plane (ESP) blocks are useful for pain relief in thoracic procedures. We are an established pleural centre offering MT [2, 6] under target-controlled anesthesia using propofol and remifentanil infusions and with the advent of the Covid-19 pandemic, we had to change our practice to offer day case MT [7] rather than admit patients afterwards for pain and pleural fluid management. For the latter, we now place an indwelling catheter in all patients rather than perform talc poudrage-the rationale behind this is out of scope of this article. We started using have been using two level, high volume ESP block pre-procedure for post-operative analgesia. A quality improvement project was devised (Reference number 7825) with local ethical approval (Northumbria Healthcare Caldicott Reference C3874) to examine patient satisfaction and pain outcomes related to the procedure with use of this protocol.

The erector spinae plane block is a regional anaesthetic technique, first described by Forero et al. [8]. Local anaesthetic solution is deposited, under ultrasound guidance, within the fascial plane beneath the erector spinae muscles, with the aim of anaesthetizing the thoracic spinal nerves and producing analgesia of the skin, subcutaneous tissues and viscera in this region. The mechanism of action has been hypothesized to occur through spread to the paravertebral space and thoracic spinal nerve roots. It is straightforward to perform, with a low risk of complications due to significant distance from important structures such as pleura or major blood vessels, and injection at a single site can be expected to cover multiple dermatomes through spread of local anaesthetic. Volumes and thoracic spinal levels were chosen at the discretion of the anaesthetist in accordance with their usual practice (adjusted according to the weight of the patient) after discussion with the respiratory physician and confirmation of likely area of port site (confirmed with point of care thoracic ultrasound). This is depicted in Figure 1 (reproduced from Ayub et al. [9] – Figure under a Creative Commons license)

**Figure 1:**
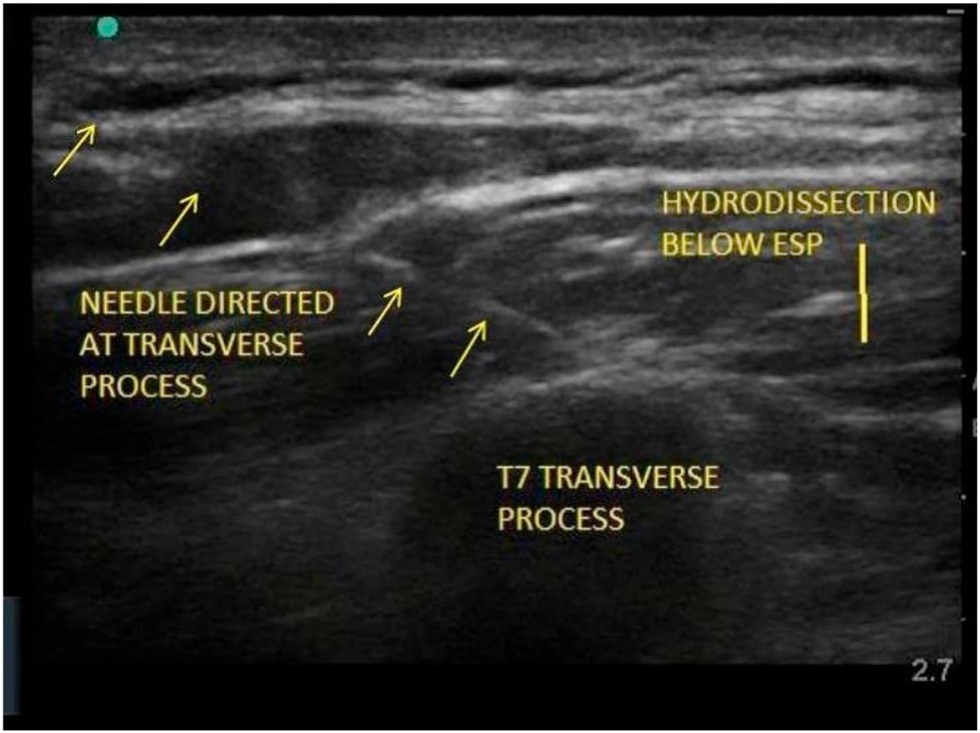
Figure showing needle aiming for the traverse process and showing the hydrodissection figure used under a creative commons licence by Ayub et al. [9].

Nine patients underwent MT from Sept 2021 to Feb 2022. Local anaesthetic use, peri-and post-operative opioid use and depth of required sedation was recorded. Pain scores during procedure, in recovery and at home were recorded by an investigator-led functional pain questionnaire (described in case record form-Appendix 1) administered directly to the patient 1-h post procedure and via telephone 24 and 48 h post procedure.

Five patients had single site ESP blocks with injections at spinal levels ranging between T5-7, using 20–35 mL total volume of 0.25% l-bupivacaine. Four patients had bi-level ESP blocks at T4 and 8, using 34–40 mL total volume of 0.25% l-bupivacaine. The average greatest depth of sedation required using propofol target controlled infusion was 1.92 (nanograms per milliliter) µg/mL (standard error of mean [SEM] 0.27), with remifentanil 2.52 μg/mL (SEM 0.46). 78% of patients required oral analgesia on day 0 post discharge (paracetamol and one instance of codeine with ibuprofen). A total of 55% of patients required oral analgesia on the postoperative day 1. Patients used an average of 3.33 mg oral morphine (SEM 2.35) in hospital, and 3 mg (SEM 2) on the post-operative day 1. Periprocedural pain scores (using the numerical rating scale, one least amount of pain and 10 being the worst pain) were 0.66 (SEM 0.27). Pain scores in the recovery room were 1.56 (SEM 0.76). Pain scores 3–12 h post discharge were 3.56 (SEM 0.7), while pain scores on the first postoperative day were significantly higher at 5.56 (SEM 0.90) (Figure 2). Functional pain scoring showed that overall patients were able to tolerate activities of daily living well on discharge and reported a good ability to breathe and cough, but felt less able to perform activities such as light housework or heavier exercise (Figure 3). There was a non-significant trend towards greater levels of function overall on post-op day 0 than on day 1. A total of 100% of patients felt that overall their pain was well controlled on the day of the procedure and after returning home. No complications of the block were reported.

**Figure 2:**
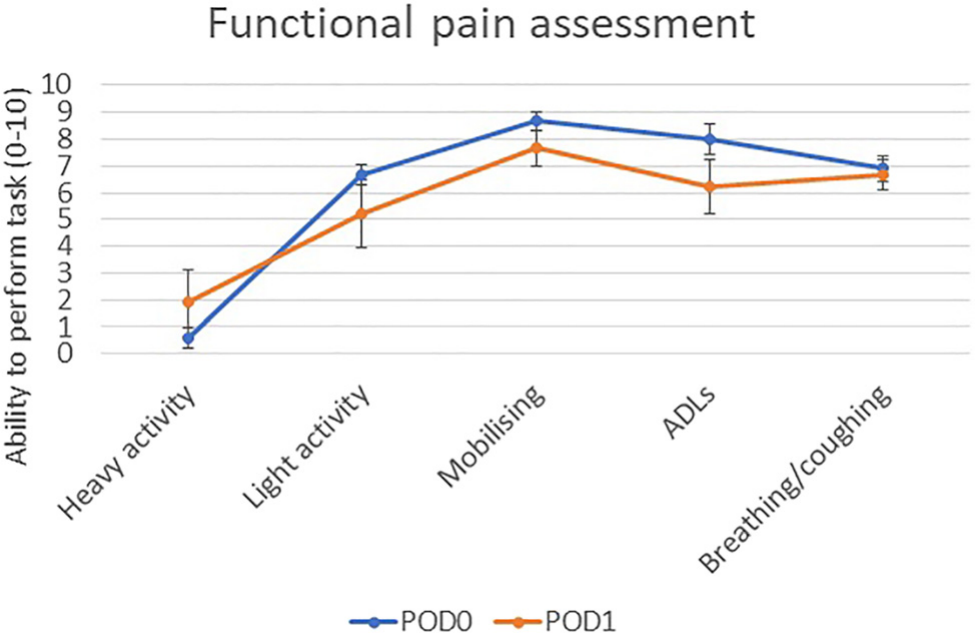
Pain scores over time following procedure assessed by telephone questionnaire (n=9).

**Figure 3:**
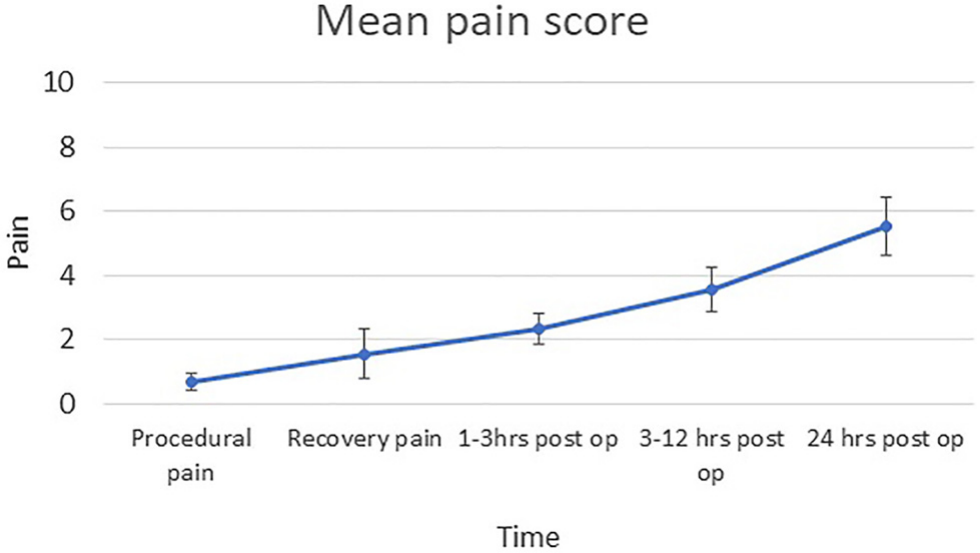
Functional pain scoring following discharge from hospital (n=9).

ESP blocks thus appear to provide good peri-procedural analgesia for MT requiring simple oral analgesia or weak opioids after discharge, and are well tolerated with patient satisfaction. Pain scores increased over time in keeping with a significant analgesic effect lasting several hours from the ESP blocks then wearing off. Regional anesthesia is known to prevent chronic pain after surgery [9] and thoracic procedures are known to carry a significant risk of persistent postoperative pain [10]: therefore, this regional anaesthetic technique shows promise for further work. It is not possible to 0ascertain the likely effect of this technique as we lack a control group but the technique is safe and can be applied. There has been some preliminary work on improving pre-procedural analgesia in MT, and this builds on the potential value of ESP being used in MT. Midpoint transverse process to pleura blocks have been described [11] as well as multi-level intercostal nerve blocks [12] and paravertebral blocks [13]. Intracavitary anesthesia has also been trialed via a small single centre pilot study, with no difference in pain scores between the two groups [14]. All these single centre studies are limited by the same factors as our single centre project: the impossibility of generalization, lack of appropriate randomization, small patient numbers but taken together, they form the basis for the creation of a large formal randomised controlled trial with multiple analgesic arms with patient related outcome measures. This is currently being planned locally and we hope that in the months to come, we will be able to publish a protocol and extend expression of interests to various multinational centers. One of the further limitations of this might be the lack of availability of anaesthetic colleagues to perform the ESP. However, locally, respiratory physicians have started to learn to perform this technique. The potential for further service development is thus immense.

## Supplementary Material

Supplementary Material
